# The Influence of Clustered DNA Damage Containing Iz/Oz and ^OXO^dG on the Charge Transfer through the Double Helix: A Theoretical Study

**DOI:** 10.3390/molecules29122754

**Published:** 2024-06-09

**Authors:** Bolesław T. Karwowski

**Affiliations:** DNA Damage Laboratory of Food Science Department, Faculty of Pharmacy, Medical University of Lodz, ul. Muszynskiego 1, 90-151 Lodz, Poland; boleslaw.karwowski@umed.lodz.pl

**Keywords:** clustered DNA damage, 7,8-dihydro-8-oxo-2′-deoxyguanosine (^OXO^dG), 2,5-Diamino-4*H*-imidazol-4-one (Iz), 2,2,4-triamino-5(2*H*)-oxazolone (Oz), BER, MutYh, charge transfer, ionization potential, electron affinity

## Abstract

The genome—the source of life and platform of evolution—is continuously exposed to harmful factors, both extra- and intra-cellular. Their activity causes different types of DNA damage, with approximately 80 different types of lesions having been identified so far. In this paper, the influence of a clustered DNA damage site containing imidazolone (Iz) or oxazolone (Oz) and 7,8-dihydro-8-oxo-2′-deoxyguanosine (^OXO^dG) on the charge transfer through the double helix as well as their electronic properties were investigated. To this end, the structures of *oligo-Iz*, d[A_1_Iz_2_A_3_^OXO^G_4_A_5_]*d[T_5_C_4_T_3_C_2_T_1_], and *oligo-Oz*, d[A_1_Oz_2_A_3_^OXO^G_4_A_5_]*d[T_5_C_4_T_3_C_2_T_1_], were optimized at the M06-2X/6-D95**//M06-2X/sto-3G level of theory in the aqueous phase using the ONIOM methodology; all the discussed energies were obtained at the M06-2X/6-31++G** level of theory. The non-equilibrated and equilibrated solvent–solute interactions were taken into consideration. The following results were found: (A) In all the discussed cases, ^OXO^dG showed a higher predisposition to radical cation formation, and B) the excess electron migration toward Iz and Oz was preferred. However, in the case of *oligo-Oz*, the electron transfer from Oz_2_ to complementary C_4_ was noted during vertical to adiabatic anion relaxation, while for *oligo-Iz*, it was settled exclusively on the Iz_2_ moiety. The above was reflected in the charge transfer rate constant, vertical/adiabatic ionization potential, and electron affinity energy values, as well as the charge and spin distribution. It can be postulated that imidazolone moiety formation within the CDL ds-oligo structure and its conversion to oxazolone can significantly influence the charge migration process, depending on the C2 carbon hybridization *sp^2^* or *sp^3^*. The above can confuse the single DNA damage recognition and removal processes, cause an increase in mutagenesis, and harm the effectiveness of anticancer therapy.

## 1. Introduction

**Figure 1:**
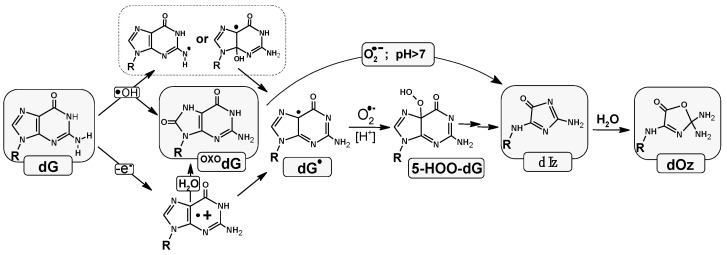
Graphical representation of imidazolone formation in the physiological condition and its conversion into oxazolone as the final product of four-electron guanine oxidation.

Across all species, each and every cell contains genetic information encoded in the sequence of nucleobases. The human genome comprises approximately 3.2 × 10^9^ base pairs (BPs) arranged in two complementary counter-rotating oligonucleotide chains settled as chromatin in the nucleus [1]. The mitochondrial circular genome, on the other hand, consists of only 16 569 BP units [2]. The above should be multiplied by the number of human body cells, i.e., 10^14^ [3]. Each of the cells, including those located within nucleic acids, is continuously exposed to harmful chemical and physical external factors, such as ionization radiation (X-ray, gamma, and beta), ultraviolet, photosynthesis, xenobiotics, environmental pollution, and reactive oxygen or nitrogen species (ROS or RNS). Additionally, other classes of potentially toxic agents exist within cells, including metabolites, products of the respiratory cycle, and estrogens [4]. The activity of all these factors causes the formation of various types of DNA damage. To date, more than 70 different kinds of DNA lesions have been identified, including apurinic-apyrimidinic sites (AP-sites), DNA-DNA or protein–DNA cross-links, single/double-strand breaks (S/DSB), sugar/base modifications, and tandem and clustered types [5]. Such DNA damage can constitute serious threats to genetic information. As a consequence, throughout evolution, prokaryotic and eukaryotic life forms have developed DNA damage response (DDR) systems that exploit base and nucleotide excision repair (BER and NER), mismatch repair, homologous recombination, and non-homologous end-joining systems, all of which remove lesions in a substrate-dependent manner [6]. Due to the abundance of sugar and base lesions which are removed from the genome by BER, this protective system has become the most active and common, which is present in the nucleus and mitochondria [7]. The protein deficit/defect involved in this repair process causes changes in genetic information (i.e., mutation), which can lead to cancers or an acceleration in the aging process [8]. Activation of the BER enzyme cascade is triggered when a DNA lesion is recognized by mono- or bifunctional glycosylase [9]. Of all the nucleosides, 2′-deoxyguaosine (dG) is especially sensitive to harmful effects because of its particularly low reduction potential among canonical nucleosides (*E*_7_(dG(-H)•/dG = 1.29 V) [10,11,12]. The lesions derived from guanosine have been identified as the most abundant. Their distribution depends on the endocellular environment (hypoxic or normoxic), the presence of a 2-deoxyribose substituent, and the ways in which ROS are generated, especially hydroxyl radicals (*●*OH) [13,14]. In a recent study using the high-salt DNA extraction method, 7,8-dihydro-8-oxo-2′-deoxyguanosine (^OXO^dG)—the most abundant DNA lesion—was measured to be 14.62 ± 1.45 per 10^6^ DNA bases [15]. The redox potential of ^OXO^dG was found to be 0.58–0.75 V [16]. This lesion is recognized and removed from the genome by the bifunctional glycosylase OGG1 [17]. A lack or defect in this enzyme leads to GC→TA transversion, because ^OXO^dG, after adopting an *anti*-conformation, can form a base pair with 2′-dexyadenosine (dA) [18]. Fortunately, the glycosylase MutYh is able to recognize and remove the badly paired adenine [19]. All the above leads to the low mutagenicity of the abundant 7,8-dihydro-8-oxo-2′-deoxyguanosine. The above-mentioned ^OXO^dG can be produced in the genome not only by *●*OH activity but also as the product of the two-electron dG oxidation process [20]. The dG and ^OXO^dG also undergo conversion to 2,5-Diamino-4*H*-imidazol-4-one (Iz), which, after hydrolysis, rearranges itself as 2,2,4-triamino-5(2*H*)-oxazolone (Oz) (Figure 1) [16,21,22].

It should be pointed out that Iz and therefore Oz are the results of the 2′-deoxyguanosine four-electron oxidation process. Moreover, Oz, and therefore Iz, leads to GC→CG or GC→TA transversion [23]. Careful analytical studies have revealed that there are 2–6 oxazolone molecules per 10^7^ of guanines in human hepatocytes [16,24].

It should be pointed out that Oz is at least one magnitude more promutagenic than ^OXO^G [25,26]. Fortunately, this lesion is removed from the genome by Nei, Nth prokaryotic glycosylases, and by eukaryotic NEIL1, NTH1 glycosylases [27,28].

Recently, a theory has been proposed that some proteins utilize a charge transfer to scan the double helix to detect their sites’ action in a highly effective way [29]. Proteins containing [4Fe-4S] clusters, such as glycosylases, polymerases, helicases, and primases, are communicated by charge transfer through ds-DNA [30]. Hence, the formation of clustered DNA lesions containing ^OXO^dG and Iz/Oz can pose a challenge for the activity of such an enzyme in the sea of canonical nucleobases. In light of the influence of clustered DNA lesions (CDLs) on the ds-DNA structure, their electronic properties and role in charge transfer are worth investigation. (A CDL is defined as two or more DNA damage events per one or two double helix turns [31,32]**.**) The above has led to a better understanding of the roles of CDLs in genetic information repair and replication [33,34]. Furthermore, the effectiveness of radiotherapy, photodynamics, chemotherapy, etc., relies on inducing DNA damage in the targeted genome. Therefore, studying the charge transfer process through the double helix should result in the increased safety of anticancer therapies (by reducing genotoxicity in normal cells) [31,34,35].

## 2. Results

**Figure 2:**
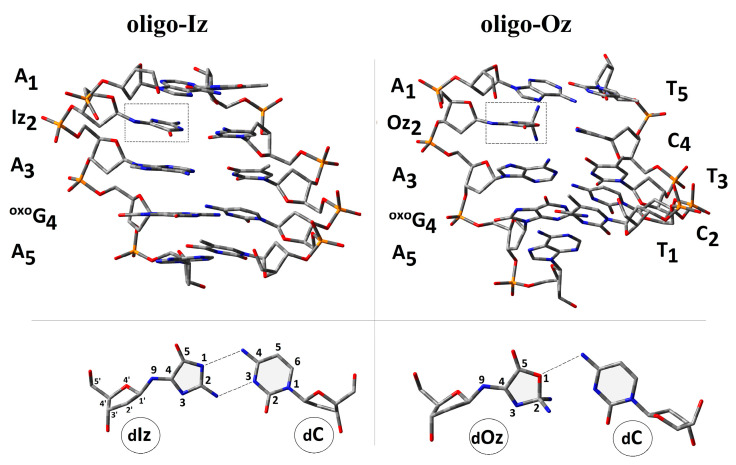
A graphical representation of the d[A_1_Iz_2_A_3_^OX**O**^G_4_A_5_]*d[T_1_C_2_T_3_C_4_T_5_] and d[A_1_Iz_2_A_3_^OX**O**^G_4_A_5_]*d[T_1_C_2_T_3_C_4_T_5_] spatial geometry optimized at the M06-2x/D95** level of theory in the aqueous phase using the PCM solvation model. Iz: 2-amino-5-[2-deoxy-*β*-d-*erythro*-pentofuranosyl)amino]-4*H*-imidazol-4-one; Oz: (2-deoxy-β-d-erythro-pentofuranosyl)amino]-5(2H)-oxazolone; and ^OX**O**^G: 7,8-dihydro-8-oxo-2’-deoxyguanosine. Extracted from the double helix base pair formed by dIz/dOz and 2’-deoxycytidine (dC) as well as the atom numbering and hydrogen bond indicated by the dash lines.

Nucleobases present in ds-DNA interacted mutually by the hydrogen bond and stacking interaction equally [36]. These gentle interactions stabilized the ds-DNA structure and, therefore, genetic information. Moreover, the presence of the *π-π* interaction facilitated the protein (containing a [4Fe-4S] cluster) communication by charge transfer (CT), e.g., glycosylases [37]. The proposed mechanism is evidence of the high effectiveness of the ds-DNA scanning/verification process by dedicated proteins [38]. Spatial geometry disruption, triggered by DNA damage, infected the CT and “activated” the glycosylase action. For these studies, short ds-oligonucleotides (ds-oligos) containing a multi-damage site consisting of ^OXO^dG and Iz or its degradation product, Oz, were investigated (Figure 2).

The following notation of the ds-oligonucleotides has been used within this article: *oligo-Oz:* d[A_1_Iz_2_A_3_^OXO^G_4_A_5_]*d[T_5_C_4_T_3_C_2_T_1_], and *oligo-Oz*: d[A_1_Oz_2_A_3_^OXO^G_4_A_5_]*d[T_5_C_4_T_3_C_2_T_1_]. As a reference for the presented studies, the unmodified ds-oligonucleotide d[A_1_G_2_A_3_G_4_A_5_]*d[T_5_C_4_T_3_C_2_T_1_] (*oligo-N*) was chosen [39].

### 2.1. Influence of Iz and Oz on Double Helix Spatial Geometry

The influence of a CDL containing ^OXO^dG and Iz or Oz on the double helix three-dimensional geometry was taken into consideration. The spatial structure of both *oligo-Iz* and *oligo-Oz* were optimized using the ONIOM (Our Own N-layered Integrated Molecular Orbital and Molecular Mechanics) methodology [40]. The central/pivotal part of the *ds*-oligo containing the complementary nucleobases was described as a high layer, while the sugar-phosphate backbone was noted as low and optimized at the M06-2x/D95** and M06-2x/sto-3G levels of theory, respectively. All the theoretical experiments were performed in the aqueous phase using the integral equation formalism variant of the polarizable continuum model (IEF-PCM) [41,42]. According to the standard reference frame for the description of DNA double helix geometry, an analysis of the mutual base arrangement was carried out for *oligo-Iz* and *oligo-Oz* [43]. For the proposal under discussion, the hydrogen bond length (HB), the distance between C1′ (*d*_C1…C1′_) of the complementary BP and rotation of each base around the C1′ atom, i.e., *λ_1_* and *λ_2_* as angles determined by N9-C1′ (pyrimidine)-C1′(purine) and N1-C1′(purine)-C1′(pyrimidine) (Table S1), have been discussed.

The analysis of the spatial geometry obtained for *oligo-Iz* and *oligo-Oz* reveals that Iz can form two hydrogen bonds with complementary cytidine, while oxazolone forms only one in its cyclic form, as presented in Figure 1. For the remainder of the BPs, the scheme of the formed HB was found to be unchanged; moreover, the lengths of the HBs were at a similar level to those noted in the standard reference frame [43]. The mutual base orientation within the BP was defined by the *λ*_1_, *λ*_2_, and *d_C1′…C1′_* parameters. A significant divergence was noted in the part of *ds*-oligo in which Iz and Oz appear. For *oligo-Iz*, the decreases in *λ*_1_ for the Iz_2_C_4_ and the neighboring A_3_T_3_ were noted as follows: 42.5[O] and 48.7[O], respectively; subsequently, a distance *d_C1…C1_* increase by 0.7 Å in the case of Iz_2_C_4_ was observed (Table S1 in the Supplementary Materials). The subsequent imidazolone hydrolysis led to oxazolone formation and caused further *λ_1_* decreases (39.6[O]); surprisingly, the *λ*_1_ of A_3_T_3_ reconstructs close to the value of the native AT base pair (52.8[O]). Additionally, the changes in *d_C1′…C1′_* of A_1_T_5_ and Oz_2_C_4_ were noted as follows: decreases by 1 Å and 0.2 Å, in comparison to those assigned for the precursor (*oligo-Iz*). In all the discussed cases, *λ*_2_ calculated for the pyrimidine strand was found to be close to the reference value of 54.5[O].

The above-discussed parameter changes influence the distance within the base-pair dimer, noted as *Rise* (Table S1) [43]. The calculated *Rise* parameters reveal slight decreases in comparison to the reference (*oligo-N*), with the average values assigned as 2.96 Å and 3.12 Å for *oligo-Iz* and *oligo-Oz*, respectively. It should be pointed out that in the wake of Iz hydrolysis, the distance between Oz_2_T_4_ and A_3_T_3_ increases from 2.93 Å up to 3.46 Å, which can be the result of *sp^3^* hybridization adoption by carbon C2 and the appearance of an additional exocyclic NH_2_ group. All this forces a higher spatial hindrance than that observed in the case of a flat Iz heterocycle moiety (Figure 1).

### 2.2. Influence of Iz and Oz on Double Hydrogen Bonds and Stacking Energies

Two main factors determine the stability of the double helix, i.e., the stacking and hydrogen bonds [36]. These noncovalent interactions are sensitive to the changes within the nucleoside/tide subunit structure. Imidozolone and oxazolone, in fact, are the products of the four-electron guanine oxidation process [22]. As shown in Figure 1, in both cases, the rearrangement process spreads over the whole bicyclic purine moiety. This structural difference from the initial guanine caused the change in the HB and *Rise* parameters as discussed previously. These geometrical changes were reflected in the values of the HB (E_HB_) and stacking (E_ST_) energies. An analysis of the results, presented in Table S2, shows that the flat structure of Iz (*oligo-Iz*) left the stacking interaction between A_1_T_5_/A_3_T_3_ and Iz_2_C_4_ equal to 14.61 and 14.97 kcal, respectively. Decreases in the HB energy versus native G:::C system (17.23 kcal) were observed for the Iz_2_C_4_ base pair, i.e., 11.47 kcal, as a result of the reduction in HB numbers. However, it was still at the level of the E_HB_ of the AT base pair. Hydrolysis of the Iz moiety led to significant E_ST_ decreases in the Oz neighborhood, as denoted by the results obtained for *oligo-Oz*, as follows: 12.41 kcal and 12.77 kcal for A_1_T_5_||Oz_2_C_4_ and Oz_2_C_4_||A_3_T_3,_ respectively. Moreover, the Iz rearrangement toward Oz led to only one hydrogen bond remaining between Oz and the complementary cytidine. The above was manifested by an E_HB_ decrease of up to 7.69 kcal, which is lower by approximately 10 kcal, than that observed in the cases of the GC or ^OXO^GC pair (Table S2).

### 2.3. Influence of Iz and Oz on Double Helix Electronic Properties

Because of the π-π interaction of the nucleobase (heterocycles), the DNA double helix can be perceived as a nanowire [44]. Moreover, investigations have revealed that charge transfer is exploited by proteins to scan the genome for different activity [37,45]. With the above in mind, the electronic properties of *oligo-Iz* and *oligo-Oz* were investigated. As shown above, Iz and Oz change the 3D geometries of the double helix. Therefore, complete ds-DNA structures consisting of a sugar–phosphate skeleton and nucleobase pairs, and solely base pair ladders, were taken into investigation. All the energy calculations were performed at the M06-2x/6-31++G** level of theory in the aqueous phase, using the IEF-PCM model. Both non-equilibrated (NE) and equilibrated (EQ) solvent–solute interactions were used. Firstly, the spatial structure of *oligo-Iz* and *oligo-Oz* in anionic and cation forms was optimized to the ground state using the ONIOM strategy at the same level as that for the non-charged ds-oligo.

The global structural changes, forced by electron attachment or electron loss, were assigned as the differences between the atomic positions between the neutral versus anionic or cationic forms of the discussed oligo, i.e., oligo-Iz and oligo-Oz, and expressed as an RMSD (Root Mean Square Deviation) in [Å^2^] (Table 1) [46]. As expected, the molecule charge changes were compensated mainly by the phosphate-sugar backbone’s flexibility, while the internal part containing base pairs was less affected, as presented in Table 1. Furthermore, the appearance of an extra electron in the *oligo-Iz* system resulted in a higher RMSD value in all the discussed cases.

Contrary to this, the electron lost by *oligo-Iz* did not force a significant structural perturbation and left the RMSD close to 0.18 Å. Comparing the *oligo-Oz* structure in the anionic and cationic modes with the structure in a neutral mode revealed a similar geometry sensitivity to the electron adoption or ejection by the ds-DNA as follows in [Å^2^]: 0.46 and 0.69 for the *oligo-Oz* anion and cation versus neutral form, respectively (Table 1).

The observed structural changes in the double helix should influence the global electronic properties. To this end, the NE and EQ interaction between the solvent and solute was investigated. It should be pointed out here that whole short fragments of ds-oligos were submerged in the aqueous phase. The results obtained for the vertical ionization potential (VIP) calculated in the non-equilibrated mode were found, respectively, to be *oligo-N*>*oligo-Oz>oligo-Iz*, irrespective of the investigated system, i.e., whether a complete double helix or base-pair skeleton. Moreover, the observed order of the VIP^NE^ remained unchanged for the equilibrated VIP and adiabatic ionization potential (AIP) (Table 1) [39]. However, the values of the discussed parameters found for *oligo-Iz* and *oligo-Oz* were at the same level in comparison to the native *oligo-N*. These indicated that the presence of ^OXO^G in ds-DNA exerts a stronger influence on radical cation formation/stabilization than Iz or Oz. The ionization potential decreases with the progress of the discussed ds-oligo system relaxations, i.e., VIP^NE^ > VIP^EQ^ > AIP.

The situation changes when the extra electron appears in the ds-DNA structure. The *oligo-Iz* in all the discussed cases had a significantly higher electron affinity than *oligo-Oz* and the reference *oligo-N*, as shown in Table 1. After structural relaxation, the following order of AEA (adiabatic electron affinity) was found for the complete ds-DNA and BP skeleton as follows: *oligo-Iz* > *oligo-N* > *oligo-Oz*. Interestingly, the AEA value established for *oligo-Oz* was lower than that obtained for *oligo-N* in both modes. Moreover, the VEA^EQ^ and VEA^NE^ were found to be completely incoherent and depend on the investigated system, i.e., a complete double helix or base-pair ladder (Table 1). These observations indicate that the presence of Oz in the ds-oligo structure, as a part of a CDL, causes significant “electro-negativity” perturbation and “covers” the influence of ^OXO^G.

As shown above, the CDL appears in the ds-DNA structure, forcing different results depending on the four-electron oxidation product, i.e., imidazolone or oxazolone in the global context. To shed light on this phenomenon, the electronic properties of isolated base pairs were taken into consideration. The above allows for the AIP, VIP, VEA, and AEA values to be obtained for the BPs in their geometry adopted in the neutral and charged (anionic and cationic) *oligo-Iz* and *oligo-Oz*, calculated at the M06-2x/6-31++G** level of theory in the condensed phase, using the equilibrated mode of solvent–solute interaction. In all the discussed cases, the lower vertical/adiabatic ionization potentials in [eV] were found for ^OXO^G_4_C_2_ as follows: 5.94/5.52 (*oligo-Iz*) and 5.91/5.56 (*oligo-Oz*), respectively (Table 2). On the other hand, a higher VIP and AIP were noted for the Iz_2_/C_4_ and Oz_2_/C_4_ base pairs at a level of 7 eV, with a negligible difference between the vertical and adiabatic states.

For the remaining A_1_T_5_, A_3_T_3_, and A_5_T_1_ base pairs present as previously in the *oligo-Iz* and *oligo-Oz* structures, the difference between the VIP and AIP was found to be negligible in a range of between 6.63 and 6.73 [eV]. The appearance of an extra electron within the ds-DNA structure leads to anion formation. The ability of electron adoption by the isolated nucleobase was described by the electron affinity parameters calculated in the vertical and adiabatic modes at the same level of theory as the ionization potential (Table 2). The highest VEA and AEA was found for the Iz_2_C_4_ moiety (*oligo-Iz*), i.e., 2.45 and 2.97 [eV], respectively, while for the Oz_2_C_4_ base pair located in *oligo-Oz*, these values were as follows: 1.82 and 1.89 [eV]. For the remaining A_1_T_5_, A_3_T_3_, A_5_T_1_, and ^OXO^G_4_C_2_, the difference between the VIP and AIP was found to be scant for both the discussed ds-oligos. The average value for the AT base pairs was found to be, in [eV], 1.42 and 1.40, respectively, while for ^OXO^GCs this value was measured as 1.51 [eV] for both the VEA and AEA (Table 2).

The above results indicate the differentiation in the spin and charge distribution within the *oligo-Iz* and *oligo-Oz* structures. To investigate this phenomenon, all the calculations were carried out at the M06-2x/6-31++G** level of theory using the Hirshfeld population analysis of the density functional electronic charge distribution [47]. As could be expected, the electron lost by *oligo-Iz* or *oligo-Oz* leads to a positive charge and spin accumulation, mainly on the ^OXO^G_4_C_2_ base pairs, of ~80% and ~90%, respectively, in both the discussed vertical and adiabatic modes. The above charge pattern found in the case of equilibrated and non-equilibrated solvent–solute interaction was affected too.

**Figure 3:**
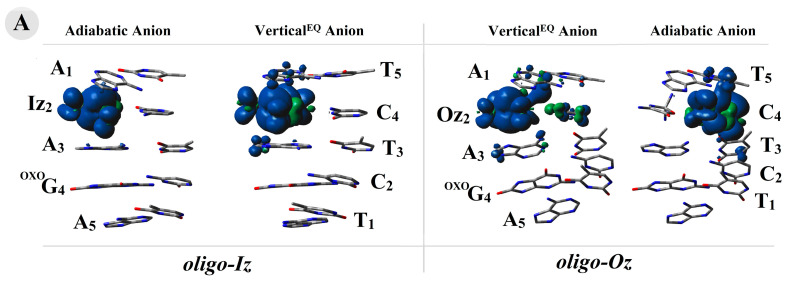

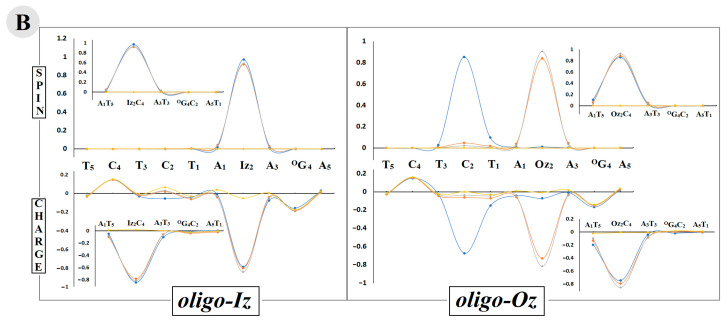
(**A**) The spin and charge distribution within oligo-Iz and oligo-Oz calculated at the M062x/6-31++G** level of theory in the condensed phase, with the base-pair skeleton taken into consideration. 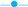 adiabatic radical anion, 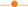 vertical radical anion, non-equilibrated solvent–solute interaction, 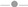 vertical radical cation/anion, equilibrated solvent–solute interaction, and 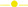 neutral form. The raw data of the charge and spin distribution have been given in Tables S3 and S4 (Supplementary Materials). (**B**) A graphical visualization of the spin distribution within *oligo-Iz* and *oligo-Oz*. The raw data have been given in Table S5.

In both ds-oligo cases, ^OXO^G was found as the main beneficiary of the positive charge and spin localization/accumulation, as presented in Table S3. In contrast, the electron adoption by the discussed systems (*oligo-Iz* and *oligo-Oz*) caused a different negative charge and spin distribution depending on whether an imidazoline or oxazolone moiety was present in the structure of the lesioned ds-oligo. In both cases (*oligo-Iz* and *oligo-Oz*), the base pairs Iz_2_C_4_ and Oz_2_C_4_ turned out to be the settling point of the extra electron (Table S3**)**. Hardly any spread of negative charge and spin was found on the A_1_T_5_ moiety. However, a more precise analysis revealed that the product of Iz heterocycle hydrolysis, i.e., Oz, exerted a significant influence on the negative charge and spin location within the double helix. Firstly, in the case of Iz_2_C_4_, the charge and spin were found in ~80% and 90% on the Iz_2_ moiety, respectively, for the vertical anion: the non-equilibrated and equilibrated state as well as for the adiabatic one (Figure 3). Secondly, the imidazolone heterocycle transformation into oxazolone caused the negative charge to settle initially (a non-equilibrated solvent–solute interaction) on the Oz (74%) and with subsequent increases up to 82% after system equilibration. A similar observation was noted in the case of the spin analysis within oligo-Oz, with 84% and 91% located on the Oz_2_ moiety in the vertical anion state, non-equilibrated and equilibrated, respectively, and barely anything at all on C_4_. A structural relaxation and achievement of the adiabatic form of the negative charge was found on the C_4_ subunit of Oz_2_C_4_ in 67% of the remaining part of the charge dispersed along the purine strand. The above is in good agreement with the spin distribution, which was located in 85% on the C_4_ base and only in 10% on T_5_, as shown in Figure 3.

### 2.4. Influence of Iz or Oz on Charge Transfer through oligo-Iz and oligo-Oz

The double helix’s ability to transfer charge follows directly from the base-pair stacking interaction [48]. Since the 1990s, ds-oligo has been perceived as a nanowire [49]. As discussed previously, the ds-DNA structure and electronic properties are influenced by the presence of Iz and Oz as part of a CDL. The additional electron or electron-hole migration process can be perceived according to three categories: single-step tunnelling, random-walk multistep, and polaron-like hopping [50]. It has been confirmed theoretically and experimentally that the charge can migrate over 200 Å within ds-DNA from the place of induction via an incoherent mechanism [51]. Contrary to this, a single-step super exchange process over a distance of a few BPs can be observed. In the case of both mechanisms, the base pairs’ spatial orientation and their individual electronic properties are essential. The theory proposed by Marcus takes all the above-discussed factors together with the rate constant (*k_ET_*) equation, as presented below:

$$k_{ET}=\frac{4\pi {\left|V_{12}\right|}^{2}}{h^{-}}\sqrt{\frac{\pi }{4\pi \lambda k_{b}T}}\cdot \exp\left(\frac{-{\left(\Delta G+\lambda \right)}^{2}}{4\pi \lambda k_{b}T}\right)$$

(*k_b_*—the Boltzmann constant, *h*—the Planck constant, *T*—the temperature [K], *λ*—the reorganization energy [eV], Δ*G*—the driving force [eV], and *V*_12_—the electron coupling) [52,53,54]. For details, please see Voityuk’s review [55].

The charge transfer rate depends on the following parameters: the driving force Δ*G* (the free energy difference between the initial and final molecule states involved in charge transfer) and the reorganization energy (*λ*) (the changes in adjacent structures, which occur during hole or electron migration). Both of the above are linked together in the activation energy (*E_a_*), which depends on the intervening system [56]. Due to the nature of the charge transfer, only the process with negative *ΔG* was taken into consideration (Table 3). Higher |Δ*G*| electron-hole (positive charge) migration values were found for A_3_T_3_→^OXO^G_4_C_2_ and ^OXO^G_4_C_2_←A_5_T_1_ in *oligo-Iz* and *oligo-Oz* at a level of close to 1.1 [eV]. Meanwhile, for A_1_T_5_←Iz_2_C_4_ and Iz_2_C_4_→A_3_T_3_ of *oligo-Iz* and A_1_T_5_←Oz_2_C_4_;Oz_2_C_4_→A_3_T_3_ of *oligo-Oz*, this value was found to be almost two and half times smaller, in the range of 0.3–0.4 [eV]. The calculated *k_ET_* for A_3_T_3_→^OXO^G_4_C_2_ and ^OXO^G_4_C_2_←A_5_T_1_ was found as follows, in [s^−1^]: 10^6^; 10^10^ and 10^9^ for *oligo-Iz* and *oligo-Oz*, respectively. Hence, negative reorganization values and activation energies were noted in the case of electron-hole transfer from the A_1_T_5_ toward the Iz_2_C_4_ and Oz_2_C_4_ base pair (A_1_T_5_→Oz_2_/Iz_2_C_4_) for both the discussed ds-oligos and Iz_2_C_4_→A_3_T_3_ for *oligo-Iz*. On the other hand, an analysis of the *ΔG* values indicates that the radical cation migration from Iz_2_C_4_ toward ^OXO^G_4_C_2_ and Oz_2_C_4_ toward ^OXO^G_4_C_2_ is privileged, i.e., *k_ET_* = 10^4^ and 10^3^ [s^−1^] in the case of *oligo-Iz* and *oligo-Oz*, respectively (Table 3). The raw data have been given in Table S6a,b.

It should be pointed out that in the case of *oligo-Oz*, the hole transfer from A_1_T_5_ to A_3_T_3_ has the highest value, as expected. Additionally, the main spin density was found on the ^OXO^G_4_C_2_ moiety of the discussed ds-oligo in the adiabatic cation state. Based on the above and because ^OXO^G_4_C_2_ has the lowest ionization potential, this base pair can be assigned as the radical cation sink during electron-hole transfer.

This is in good agreement with previous observations. Because of its nature, the charge transfer through ds-DNA may occur in its oxidative and reduced state. The highest rate constant of the excess electron transfer was found for A_3_T_3_ → ^OXO^G_4_C_2_ of *oligo-Iz* and ^OXO^G_4_C_2_ ← A_5_T_1_ of *oligo-Oz* as follows: 7.1 × 10^11^ and 1.44 × 10^14^, respectively, in [s^−1^]. However, because of the highest absolute value of the ΔG and spin distribution results, it can be predicted that excess electron transfers are privileged toward Iz_2_C_4_ and Oz_2_C_4_ moieties within *oligo-Iz* and *oligo-Oz*. For the above, the high-rate constant value was calculated for excess electron migration toward the Iz_2_C_4_ or Oz_2_C_4_ base pair from the neighboring pairs, as presented in Table 3. Additionally, a difference in the transfer predisposition between Iz_2_C_4_/Oz_2_C_4_ and ^OXO^G_4_C_2_ was observed. In the case of *oligo-Iz*, it was Iz_2_C_2_←^OXO^G_4_C_4_ (k_ET_ = 1.39 × 10^9^ s^−1^), while in the case of *oligo-Oz*, it was noted as 8 orders of magnitude slower Oz_2_C_4_←^OXO^G_4_C_2_ (k_ET_ = 4.5 × 10^1^ s^−1^). The above is supported by the results of the adiabatic electron affinity and the negative charge and spin distribution calculation.

## 3. Discussion

The genome (ds-DNA) is continuously exposed to various extra- and intra-cellular harms [4,57]. Under suitable conditions, their activity causes different types of DNA damage. Until now, more than 70 lesion types have been identified [5,58]. Therefore, the correct sequence of nucleobases in the genome is governed by different DNA repair systems, such as BER, NER, NHEJ, HR, etc. [59]. Any deficiency can lead to mutations and, as a consequence, to carcinogenesis or an accelerated aging process [60]. Most of the DNA damage is removed from ds-DNA by BER machinery [61]. The subsequent cascade of protein activity is initiated by glycosylases [62,63]. These specific enzymes identify and remove the designated lesion. However, the above process raises the important question of how these proteins are capable of scanning the genome in such an effective way so as to prevent genetic information from being permanently altered. Moreover, the accumulation of unrepaired units in the genome can cause multiple damage sites to form and subsequently affect further repairs to lesions [64]. Cappelli at al. have shown that the abundant glycosylases (10^5^) present in the nucleus must scan approximately 7 × 10^4^ base pairs per individual [65]. Additionally, in a single cell, 10^4^ DNA lesions are induced daily [3]. Recently, Barton et al. have shown that glycosylases like MutY can recognize a DNA lesion by charge transfer, making this process extremely fast and effective in the case of isolated lesions [37,66,67]. However, the situation becomes more complicated when a multi-DNA damage site is taken into consideration. Given the above, the influence of CDLs containing Iz or Oz and ^OXO^dG on the electron or hole transfer process was considered worthy of investigation. With this in mind, the following ds-oligos were chosen: *oligo-Iz*: d[A_1_Iz_2_A_3_^OXO^G_4_A_5_]*d[T_5_C_4_T_3_C_2_T_1_] and *oligo-Oz*: d[A_1_Oz_2_A_3_^OXO^G_4_A_5_]*d[T_5_C_4_T_3_C_2_T_1_]. The ^OXO^dG is established as one of the more abundant one-electron oxidation lesions, while imidazolone or oxazolone are less propagated four-electron oxidation examples [68]. It should be pointed out that Oz can appear in the genome as the second product of 2′-deoxyguanosie or 7,8-dihydro-8-oxo-2′-deoxyguanosine degradation [23,25]. Meanwhile, Iz can be perceived as intermediate, which after slow hydrolysis (147 min) is converted to oxazolone [23] (Figure 1). Moreover, Ming et al. have shown that the number of both lesions, i.e., ^OXO^dG and Oz, increases in the presence of 5-methycytosine when a CpG island of *p53* is taken into consideration [69]. Therefore, the discussed type of CDL on charge transfer should not be omitted. The results presented in this manuscript reveal that imidazolone forms two hydrogen bonds with complementary cytidine, while within the Oz_2_:C_4_ base only one is found. The following *E_HB_*s were noted for Iz::C and Oz:C, respectively: 12.8 and 7.7 kcal. Moreover, the presence of the final guanine four-electron oxidizing product within *oligo-Oz* impaired the stacking interaction energy of the Oz_2_C_4_||A_3_T_3_ BP dimer to 12.77 kcal, which is in good agreement with the *Rise* parameter increases of up to 3.46 Å (Tables S1 and S2). Surprisingly, when *oligo-Iz* was taken into consideration, these parameters were found at the level assigned for native *oligo-N* as follows: *Rise* = 2.93 Å and *E_ST_* = 14.97 kcal. The appearance of an additional electron within the *oligo-Iz* leads to greater structural changes than those noted for *oligo-Oz*, while the electron loss by the system leads to the opposite observation, with a greater geometry susceptibility to a positive charge being noted for *oligo-Oz*, which is shown in Table 1 as the results of RMSD value calculations. The analysis of the calculated ionization potential and electron affinity parameters of ds-oligo showed the highest AEA for *oligo-Iz* (2.83 eV), compared to *oligo-Oz* (2.06 eV), while the AIP was found at the same level (5.47 eV) in both cases. The above indicates a greater influence of Iz and Oz on electron affinity than ^OXO^G if both lesions are part of a CDL structure. Conversely, ^OXO^G determines the ionization properties of the discussed ds-oligo. The above is in good agreement with the results of the charge and spin distribution analysis performed in both the vertical and adiabatic modes. The radical cation is mainly located at the ^OXO^dG_4_C_2_ base pair, irrespective of the presence of a second DNA lesion (Iz or Oz). Conversely, the adoption of an extra electron by *oligo-Iz* and *oligo-Oz* leads to similar outcomes. The negative charge and spin were mainly found on Oz_2_C_4_ and Iz_2_C_4_. However, an advanced analysis revealed that the extra charge is mainly located on the Iz moiety in the vertical and adiabatic anion states of *oligo-Iz*. On the other hand, in the case of *oligo-Oz*, the extra electron (vertical anion) is initially located on the Oz_2_ subunit and subsequently migrates to the complementary cytidine (C_4_) base moiety. These results correspond well with the assigned electronic properties for individual base pairs extracted from a ds-oligo. For both *oligo-Iz* and *oligo-Oz*, lower ionization potentials were found for ^OXO^G_4_C_2_ (~5.5 eV), while the highest electron affinity was found for Oz_2_C_4_ and Iz_2_C_4_, i.e., 2.97 and 1.89, respectively, in eV. Taking this all together, it can be predicted that the guanosine four-electron oxidation product, constituting part of a clustered lesion together with ^OXO^G, can affect the charge transfer through a double helix. An electron-hole transfer investigation, according to the Marcus [70] theory, revealed that in the case of *oligo-Iz* and *oligo-*Oz, the radical cation migration toward the ^OXO^dG_4_C_2_ base pair was privileged. However, in the case of excess electron transfer, differences in preference were observed. In both cases, i.e., *oligo-Iz* and *oligo-Oz*, the electron transfer toward the Iz_2_C_4_ and Oz_2_C_4_ moiety was noted as privileged (Table 3). Therefore, it can be concluded that in both ds-oligo cases, the ^OXO^G_4_C_2_ base pair should be privileged to a suitable radical cation formation, while the excess electron should settle at Iz_2_C_4_ of *oligo-Iz* and at Oz_2_C_4_ of *oligo-Oz*. The above observation indicates that four-electron oxidation products can significantly affect the charge migration process through the double helix. It should be pointed out that the initial imidazolone moiety is subsequently hydrolyzed to oxazolone. This can manifest itself by the slowing down of other DNA lesion recognition and removal processes, which can increase the probability of mutagenesis and subsequent pathological processes. However, with regard to anticancer therapy (radio/chemo), the presence of Iz and further Oz in the structure of clustered DNA damage can result in improved cancer treatment.

It should be noted that four-electron oxidation is a rare event under physiological conditions. However, during anticancer therapy (e.g., ionization radiation), four-electron oxidation can occur, promoting Iz and subsequently Oz formation. Identifying and quantifying secondary oxidation products of 2′-deoxyguanosine such as Iz and Oz poses serious challenges in terms of analytical techniques because of their instability and high hydrophilicity [71]. Even in low abundance, Iz and Oz (2–6 per 10^7^ [23]) can play a significant role in mutagenesis/cancerogenesis. It has been found that unlike ^OXO^dG, which yields 3% of G → T transversion, Oz results in G → C preferences [72]. Furthermore, the presence of Iz and/or Oz in the structure of the double helix can lead to a protein communication process via charge transfer [73]. It has been found that after electron loss, the binding of MutYh to ds-DNA increases a thousand-fold [19,74,75]. If no reduction takes place, it can hinder the movement of other proteins, such as OGG1. The above indicates that the cumulation of DNA damage over a short distance renders the damage repair process difficult with a longer repair time and reduced fidelity [76].

## 4. Materials and Methods

All the theoretical calculations were carried out according to previous descriptions, in brief [77]: the starting geometries of the bi-stranded *oligo-Iz* and *oligo-Oz* were built using BIOVIA Discovery Studio Visualizer v20.1.0.19295 software [78] and denoted accordingly as d[A_1_Iz_2_A_3_^OXO^G_4_A_5_]*d[T_5_C_4_T_3_C_2_T_1_] and d[A_1_Oz_2_A_3_^OXO^G_4_A_5_]*d[T_5_C_4_T_3_C_2_T_1_], respectively.

The negative charges of the phosphate groups were neutralized by the addition of protons, and the other atoms were saturated by additional hydrogen atoms. The structure optimizations of *oligo-Iz* and *oligo-Oz* were performed using the ONIOM (Our Own N-layered Integrated Molecular Orbital and Molecular Mechanics) strategy [40]. The structures of the ds-oligos were divided into high—HL (nucleobases, M06-2X/D95**)—and low—LL (sugar-phosphate backbone, M06-2X/sto-3G)—levels of calculation [79]. In the course of the ONIOM calculation, the atoms in the HL, which are bonded to atoms in the LL system, were modeled using link atoms when the computations were performed on the HL system. In the case of a carbon atom (C^LL^) from the low level being connected with a carbon atom (C^HL^) from the high-level part (C^LL^-C^HL^ bond), the C^LL^ atom was replaced by a hydrogen atom in the ensuing energy computations [80]. The graphical representation of the ONIOM layers has been presented in Figure S1, Supplementary Materials. Following Truhlar’s and Lin’s studies, the Z3 scheme was found to be the default for the charge manipulation in the link atom approach in the ONIOM calculations using Gaussian 16 software. For details of ONIOM, please see references [81,82]. All the calculations were performed in the aqueous phase. The M06-2X functional with the augmented polarized valence double-ζ basis set 6-31++G** was used for the energy calculations. For all the optimized geometries, the charge and spin analyses were achieved using the Hirshfeld methodology at the M06-2X/6-31++G** level of theory [83]. The electronic properties of the molecules were calculated as described previously [84,85]. The chosen level of theory was previously carefully investigated and compared with other DFT functionals such as M-11, ωB97-XD, and M06-L, for which the following basis sets were used: 6-31++G*, 6-31++G**, and D95** aug-cc-pVDZ. In all the tested cases, the radical anion represented the valence type, not the dipole, except for ωB97-XD/6-31++G** [86]. The influence of the basis set and DFT functionals was previously carefully investigated by Sevilla et al. for isolated nucleobases [87]. It should be pointed out that the electronic behavior of the base pairs was found to be different in comparison to the isolated base pairs due to the possibility of a charge-proton transfer. The transition dipole moment of excited states and the single point calculation at the M06-2X/6-31++G** level of theory were performed using time-dependent DFT (TD-DFT) methodology [88]. The electron coupling was calculated according to the Generalized Mulliken–Hush model [89]. The solvation–solute interaction was investigated in both non-equilibrium (NE) and equilibrated (EQ) modes [41]. All the calculations of the electronic properties, i.e., the VIP^NE^ (vertical ionization potential in the NE state), VIP^EQ^ (vertical ionization potential in the EQ state), AIP (adiabatic ionization potential), VEA^NE^ (vertical electron affinity in the NE state), VEA^EQ^ (vertical electron affinity in the EQ state), and AEA (adiabatic electron affinity), were conducted in [eV], as described previously [85]. All the above calculations were performed using the Gaussian G16 (version C.01) software suite [90].

## Figures and Tables

**Table 1:** The electronic properties, in [eV], of *oligo-Iz* and *oligo-Oz*: the vertical (VIP) and adiabatic ionization potential (AIP) and the vertical (VEA) and adiabatic (AEA) electron affinity calculated at the M062x/6-31++G** level of theory in the aqueous phase. ^(a)^ Complete double helix and ^(b)^ base-pair skeleton, NE—non-equilibrated solvent–solute interaction, EQ—equilibrated solvent–solute interaction, and * data calculated for *oligo-N* [39]. The Root Mean Square Deviation (RMSD) of the atomic positions in [Å^2^], calculated for the neutral, anionic, and cationic forms of *oligo-Iz* and *oligo-Oz*. BP—base pair, PS—phospho-sugar backbone. The raw data have been given in Table S3.

	VIP^NE^	VIP^EQ^	AIP	VEA^NE^	VEA^EQ^	AEA
*oligo-Iz*	^(a)^ 6.47	5.85	5.47	1.53	2.22	2.83
	^(b)^ 6.25	5.78	5.37	1.08	2.14	2.81
*oligo-Oz*	^(a)^ 6.52	5.89	5.48	0.95	1.49	2.06
	^(b)^ 6.36	5.82	5.40	0.48	1.58	1.78
oligo-N *	*^(a)^ 6.72	6.08	5.65	0.84	1.58	2.09
	*^(b)^ 6.48	5.98	5.58	0.60	1.34	1.90
	RMSD: Anion versus Neutral			RMSD: Cation versus Neutral		
	*ds*-DNA	BP	PS-Frame	*ds*-DNA	BP	PS-Frame
*oligo-Iz*	0.72	0.49	0.87	0.18	0.15	0.21
*oligo-Oz*	0.46	0.38	0.52	0.59	0.46	0.69
*oligo-N* *	0.17	0.16	0.17	0.36	0.29	0.42

**Table 2:** The electronic properties of the isolated base pairs from oligo-Iz and oligo-Oz: the vertical (VIP) and adiabatic ionization potential (AIP) and the vertical (VEA) and the adiabatic (AEA) electron affinity calculated at the M062x/6-31++G** level of theory in the condensed phase. The raw data have been given in Table S4.

*oligo-Iz*					*oligo-Oz*				
Base Pair	VIP	AIP	VEA	AEA	Base Pair	VIP	AIP	VEA	AEA
A_1_T_5_	6.63	6.65	1.43	1.41	A_1_T_5_	6.65	6.72	1.39	1.40
Iz_2_C_4_	7.04	7.03	2.45	2.97	Oz_2_C_4_	7.01	7.03	1.82	1.89
A_3_T_3_	6.65	6.66	1.43	1.31	A_3_T_3_	6.62	6.63	1.42	1.44
^OXO^G_4_C_2_	5.94	5.51	1.51	1.53	^OXO^G_4_C_2_	5.91	5.56	1.52	1.48
A_5_T_1_	6.73	6.69	1.43	1.43	A_5_T_1_	6.70	6.68	1.43	1.43
GC [39]	6.13	5.83	1.52	1.95	AT [39]	6.65	6.60	1.40	1.40

**Table 3:** The charge transfer parameters. ΔG—driving force, *λ*—reorganization energy, *E*_a_—activation energy, *V*_12_—electron coupling, and *k*_HT_—charge rate constant of permissible transfer between base pairs of *oli*go*-Iz* and *oligo-Oz*, calculated at the m062x/6-31++G** level of theory in the aqueous phase and given in [eV]. The arrows indicate the direction of the charge migration.

System	Electron-Hole Transfer					Graphics
*oli*go*-Iz*	*λ*	ΔG	*E*_a_	*V*_12_	*k_HT_* [s^−1^]	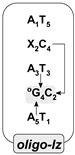
A_1_T_5_ ← Iz_2_C_4_	−0.02	−0.38	−2.04	0.22	0.00	
Iz_2_C_4_ → A_3_T_3_	−0.02	−0.37	−2.28	0.23	0.00	
A_3_T_3_ → ^OXO^G_4_C_2_	0.44	−1.15	0.28	0.43	8.49 × 10^10^	
^OXO^G_4_C_2_ ← A_5_T_1_	0.34	−1.18	0.52	0.39	7.22 × 10^6^	
A_1_T_5_ ← A_3_T_3_	−0.03	−0.01	−0.01	0.01	0.00	
Iz_2_C_4_ → ^OXO^G_4_C_2_	0.43	−1.52	0.70	0.59	1.23 × 10^4^	
A_3_T_3_ ← A_5_T_1_	−0.01	−0.03	−0.04	0.04	0.00	
*oli*go*-Iz*	Excess Electron Transfer					
A_1_T_5_ → Iz_2_C_4_	0.52	−1.56	0.52	0.37	5.73 × 10^6^	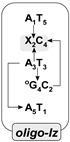
Iz_2_C_4_ ← A_3_T_3_	0.61	−1.66	0.45	0.41	8.86 × 10^7^	
A_3_T_3_ → ^OXO^G_4_C_2_	0.10	−0.22	0.03	0.01	7.10 × 10^11^	
^OXO^G_4_C_2_ ← A_5_T_1_	−0.02	−0.09	−0.16	0.05	0.00	
A_1_T_5_ ← A_3_T_3_	−0.11	−0.10	−0.10	0.07	0.00	
Iz_2_C_4_ ← ^OXO^G_4_C_2_	0.54	−1.44	0.37	0.34	1.32 × 10^9^	
A_3_T_3_ → A_5_T_1_	0.10	−0.12	0.002	0.07	2.74 × 10^14^	
*oli*go*-Oz*	Electron-Hole Transfer					
A_1_T_5_ ← Oz_2_C_4_	−0.03	−0.31	−0.88	0.21	0.00	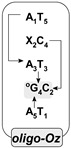
Oz_2_C_4_ → A_3_T_3_	0.02	−0.40	1.57	0.32	3.43 × 10^−11^	
A_3_T_3_→^OXO^G_4_C_2_	0.37	−1.07	0.33	0.39	9.95 × 10^9^	
^OXO^G_4_C_2_ ← A_5_T_1_	0.36	−1.12	0.39	0.39	1.04 × 10^9^	
A_1_T_5_ → A_3_T_3_	0.00	−0.09	0.49	0.02	4.54 × 10^5^	
Oz_2_C_4_ → ^OXO^G_4_C_2_	0.38	−1.47	0.76	0.59	1.22 × 10^3^	
A_3_T_3_ ← A_5_T_1_	−0.01	−0.05	−0.09	0.04	0.00	
*oli*go*-Oz*	Excess Electron Transfer					
A_1_T_5_ → Oz_2_C_4_	0.13	−0.49	0.25	0.10	2.38 × 10^10^	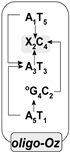
Oz_2_C_4_ ← A_3_T_3_	0.06	−0.46	0.60	0.07	1.95 × 10^4^	
A_3_T_3_ → ^OXO^G_4_C_2_	−0.04	−0.04	−0.04	0.08	0.00	
^OXO^G_4_C_2_ ← A_5_T_1_	0.03	−0.05	0.00	0.05	2.19 × 10^14^	
A_1_T_5_ → A_3_T_3_	0.07	−0.04	0.004	0.05	1.44 × 10^14^	
Oz_2_C_4_ ← ^OXO^G_4_C_2_	0.04	−0.41	0.78	0.09	4.50 × 10^1^	
A_3_T_3_ ← A_5_T_1_	0.01	−0.01	0.001	0.07	7.02 × 10^14^	

## Data Availability

Not applicable.

## Supplementary Materials

The following supporting information can be downloaded at: https://www.mdpi.com/article/10.3390/molecules29122754/s1, **Table S1**. The structural local base-pair parameters: rise and *d_C1′…C1′_* in [Å] and *λ_1_* and *λ_2_* in [O] according to the standard DNA reference frame of *oligo-Iz* and *oligo-Oz*. The hydrogen bond lengths are given in [Å] (HB-1: Ade(N1), Thy(N3) and Gua(O6), and Cyt(N4); HB-2: Ade(N6), Thy(O4) and Gua(N1), and Cyt(N3); HB-3: Gua(N2) and Cyt(O2); for Iz2C4: HB-1 (Cyt(N4), Iz(N1), HB-2 Cyt(N3), and Iz(N2); and for Oz2C4: HB-1 (Cyt(N4) and Oz(O1)). Table S2. The stacking and hydrogen bond energies in [kcal] calculated at the M062x/6-31++G** level of theory in the aqueous phase. The raw data are given in Tables S1 and S2 of the Supplementary Materials. Table S3. The energies (in Hartree) of the neural, vertical cation (VC^NC^) (NE—non-equilibrated), vertical cation (VC^EQ^) (EQ—equilibrated), vertical anion (VA^NE^), vertical anion (VA^EQ^), adiabatic cation (AC), and adiabatic anion (AA) of a complete DNA double helix and base-pair skeleton extracted from *ds*-oligonucleotides calculated at the M06-2X/6-31++G** level of theory in the aqueous phase, respectively. Table S4. The energies (in Hartree) of the neural, vertical cation, and adiabatic cation forms of the base pairs extracted from the *ds*-oligonucleotides calculated at the M06-2x/6-31++G** level of theory in the aqueous phase. Table S5. The Hirshfeld charge and spin distribution in the shape of *oligo-Iz,* d[A_1_Iz_2_A_3_^OXO^G_4_A_5_]*d[T_5_C_4_T_3_C_2_T_1_], and *oligo-Oz,* d[A_1_Oz_2_A_3_^OXO^G_4_A_5_]*d[T_5_C_4_T_3_C_2_T_1_], where only the nucleoside bases were taken into consideration, calculated at the M06-2x/6-31++G** level of theory in the aqueous phase. Vertical cation (VC^NC^) (NE—non-equilibrated), vertical cation (VC^EQ^) (EQ—equilibrated), vertical anion (VA^NE^), vertical anion (VA^EQ^), adiabatic cation (AC), and adiabatic anion (AA). Table S6a. The energies: The ground (E^GR^) and excitation (E^EX^) state energies and excitation and HOMO energies as well as the corresponding dipole moment ground, excitation, and transition (DM^G^, DM^EX^, and D_12_) in Debays of the neighboring base pair extracted from the selected dimers of the *ds*-oligonucleotides, calculated at the M06-2x/6-31++G** level of theory in the aqueous phase using the DFT or TD-DFT methodology. Table S6b. The energies: The ground (E^GR^) and excitation (E^EX^) state energies and excitation and HOMO energies as well as the corresponding dipole moment ground, excitation, and transition (DM^G^, DM^EX^, and D_12_) in Debays of the distal base pair extracted from the selected trimers of the *ds*-oligonucleotides, calculated at the M06-2x/6-31++G** level of theory in the aqueous phase using the DFT or TD-DFT methodology. Figure S1. The graphical representation of the ONIOM layer distribution used in these studies.
