# Supplementary material for: The Influence of Clustered DNA Damage Containing Iz/Oz and OXOdG on the Charge Transfer through the Double Helix: A Theoretical Study
Source: Molecules. 2024 Jun 9;29(12):2754. doi: 10.3390/molecules29122754 (PMC11206643; doi:10.3390/molecules29122754)

Supplementary Materials

The Influence of Clustered DNA Damage Containing Iz/Oz and OXOdG on the Charge Transfer through the Double Helix: A Theoretical Study

Bolesław T. Karwowski

Table S1. The structural local base-pair parameter: rise and $d_{C1'...C1'}$ in [Å], λ_1 and λ_2 in [O] according to the standard DNA reference frame of *oligo-Iz* and *oligo-Oz*. The hydrogen bonds lengths are given in [Å] (HB-1: Ade(N1), Thy(N3) and Gua(O6), Cyt(N4); HB-2: Ade(N6), Thy(O4) and Gua(N1), Cyt(N3); HB-3: Gua(N2), Cyt(O2), for Iz2C4: HB-1 (Cyt(N4), Iz(N1), HB-2 Cyt(N3), Iz(N2), for Oz2C4: HB-1 (Cyt(N4), Oz(O1)).

	Base Pair	HB_1	HB_2	HB_3	λ_1	λ_2	$d_{C1'...C1'}$	Base Pair dimmer	Rise
oligo-Iz	A_1T_5	2.85	2.93		51.5	52.0	10.7	A_1T_5 I $Iz_2\mathbf{C_4}$	2,96
	$\mathbf{Iz_2C_4}$	2.83	3.03		42.5	55.5	11.4	Iz_2C_4 I A_3T_3	2.93
	A_3T_3	2.86	2.88		48.7	50.7	10.8	A_3T_3 I $^{O}\mathbf{G_2C_4}$	2.95
	$^{O}\mathbf{G_2C_4}$	2.88	2.88	2.88	52.1	55.5	10.8	$^{O}\mathbf{G_2C_4}$ I A_5T_1	2.99
	A_5T_1	3.00	2.81		53.7	56.1	10.5		
oligo-Oz	A_1T_5	2.88	2.81		55.2	54.8	9.7	A_1T_5 I $Oz_2\mathbf{C_4}$	2.93
	$\mathbf{Oz_2C_4}$	3.02			39.6	51.2	11.2	OzC_4 I A_3T_3	3.46
	A_3T_3	2.91	2.81		52.8	52.8	10.6	A_3T_3 I $^{O}\mathbf{G_2C_4}$	2.93
	$^{O}\mathbf{G_2C_4}$	2.87	2.90	2.84	54.8	53.2	10.7	$^{O}\mathbf{G_2C_4}$ I A_5T_1	3.17
	A_5T_1	2.80	3.01		55.6	58.6	10.4		
	Parameters calculated for ideal model								
	AT*	2.96	3.05		54.5	54.5	10.7	GC I I AT**	2.96
	GC	3.00	3.00	2.87	54.2	54.5	10.8	AT I I GC	3.31
*	Olson, W.K.; Bansal, M.; Burley, S.K.; Dickerson, R.E.; Gerstein, M.; Harvey, S.C.; Heinemann, U.; Lu, X.J.; Neidle, S.; Shakked, Z.; et al. A standard reference frame for the description of nucleic acid base-pair geometry. *J. Mol. Biol.* **2001**, *313*, 229–237, doi:10.1006/jmbi.2001.4987.								
**	Karwowski, B.T. The Influence of Single , Tandem , and Clustered DNA Damage on the Electronic Properties of the Double Helix : A Theoretical Study. **2020**, 1–20								

Table S2. Stacking and hydrogen bond energies in [kcal] calculated at the M062x/6-31++G** level theory in the aqueous phase. The raw data are given in Tables S1 and S2 of the Supplementary Materials. *The reference data taken from publication.

oligo-Iz		*oligo-Oz*		*oligo-Iz*		*oligo-Oz*	
Stacking Energy (E_{ST})				Hydrogen Bond Energy (E_{HB})			
BP dimer		BP dimer		Base Pair		Base Pair	
$A_1T_5 \vert \mathbf{Iz}_2C_4$	14.61	$A_1T_5 \vert \mathbf{Oz}_2C_4$	12.41	A_1T_5	10.71	A_1T_5	10.89
$\mathbf{Iz}_2C_4 \vert A_3T_3$	14.97	$\mathbf{Oz}_2C_4 \vert A_3T_3$	12.77	$\mathbf{Iz}_2C_4$	11.47	$\mathbf{Oz}_2C_4$	7.69
$A_3T_3 \vert {}^{OXO}G_4C_2$	16.59	$A_3T_3 \vert {}^{OXO}G_4C_2$	15.59	A_3T_3	10.76	A_3T_3	11.30
${}^{OXO}G_4C_2 \vert A_5T_1$	15.42	${}^{OXO}G_4C_2 \vert A_5T_1$	15.69	${}^{OXO}G_4C_2$	17.67	${}^{OXO}G_4C_2$	18.03
AT $\vert$ GC*	13.59	AT $\vert$ ^{OXO}GC*	14.69	A_5T_1	10.59	A_5T_1	10.71
GC $\vert$ AT*	14.56	^{OXO}GC $\vert$ AT*	14.43	G:::C*	17.23	^{OXO}G:::C*	17.69
*	Karwowski, B.T. The Influence of Single , Tandem , and Clustered DNA Damage on the Electronic Properties of the Double Helix : A Theoretical Study. **2020**, 1–20						

Table S3. The energies (in Hartree) of Neural, Vertical Cation (VC^{NC}) (NE-non-equilibrated), Vertical Cation (VC^{EQ}) (EQ-equilibrated), Vertical Anion (VA^{NE}), Vertical Anion (VA^{EQ}), Adiabatic Cation (AC), Adiabatic Anion (AA) of complete DNA double helix and base pairs skeleton extracted from *ds*-oligonucleotides calculated at the M06-2X/6-31++G** level of theory in the aqueous phase, respectively

	**Neutral**	**VC^{NE}**	**VC^{EQ}**	**VA^{NE}**	**VA^{EQ}**	**AC**	**AA**
	**Complete DNA double helix**						
oligo-Iz	-12720.0095577	-12719.7946668	-12719.7946668	-12720.0909820	-12720.0909820	-12719.8085915	-12720.1134173
oligo-Oz	-12888.6634818	-12888.4475184	-12888.4475184	-12888.7766032	-12888.7766032	-12888.4616365	-12888.7955198
**Base Pairs skeleton extracted from *ds*-oligonucleotides**							
oligo-Iz	-4581.8460850	-4581.6165878	-4581.6335990	-4581.8859450	-4581.9248370	-4581.6487980	-4581.9493780
oligo-Oz	-4750.4975610	-4750.2661200	-4750.2844040	-4750.5659310	-4750.6031320	-4750.2995830	-4750.6167010

Table S4. The energies (in Hartree) of Neural, Vertical Cation, Adiabatic Cation forms of base pairs extracted from *ds*-oligonucleotides calculated at the M06-2x/6-31++G** level of theory in the aqueous phase.

oligo-Iz	**Neutral**	**Vertical Cation**	**Adiabatic Cation**
A_1T_5	-921.1922420	-920.9484330	-920.9478610
Iz_2C_4	-805.6913700	-805.4327060	-805.4328910
A_3T_3	-921.1924260	-920.9481010	-920.9476500
$^{OXO}G_4C_2$	-1012.4768740	-1012.2584190	-1012.2742360
A_5T_1	-921.1921800	-920.9448170	-920.9463270
oligo-Iz		**Vertical Anion**	**Adiabatic Anion**
A_1T_5		-921.244632	-921.244098
Iz_2C_4		-805.781444	-805.800466
A_3T_3		-921.244816	-921.240656
$^{OXO}G_4C_2$		-1012.532518	-1012.533017
A_5T_1		-921.244725	-921.244889
oligo-Oz	**Neutral**	**Vertical Cation**	**Adiabatic Cation**
A_1T_5	-921.1922720	-920.9472720	-920.9471280
Oz_2C_4	-974.3395820	-974.0797730	-974.0801080
A_3T_3	-921.1924130	-920.9492200	-920.9490470
$^{OXO}G_4C_2$	-1012.4770620	-1012.2588180	-1012.2741820
A_5T_1	-921.1921990	-920.9449970	-920.9467010
oligo-Oz		**Vertical Anion**	**Adiabatic Anion**
A_1T_5		-921.2436600	-921.2435370
Oz_2C_4		-974.4553280	-974.4679550
A_3T_3		-921.2447120	-921.2448740
$^{OXO}G_4C_2$		-1012.5329780	-1012.5326140
A_5T_1		-921.2448640	-921.2447880

Table S5. Hirshfeld charge and spin distribution in the shape of *oligo-Iz* d[$A_1Iz_2A_3{}^{OXO}G_4A_5$]*d[$T_5C_4T_3C_2T_1$] and *oligo-Oz* d[$A_1Oz_2A_3{}^{OXO}G_4A_5$]*d[$T_5C_4T_3C_2T_1$] only nucleosides bases were taken into consideration, calculated at the M06-2x/6-31++G** level of theory in the aqueous phase. Vertical Cation (VC^{NC}) (NE-non-equilibrated), Vertical Cation (VC^{EQ}) (EQ-equilibrated), Vertical Anion (VA^{NE}), Vertical Anion (VA^{EQ}), Adiabatic Cation (AC), Adiabatic Anion (AA)

oligo-Iz							
	**Neutral**	**VC^{NE}**		**VC^{EQ}**		**AC**	
	**Charge**	**Charge**	**Spin**	**Charge**	**Spin**	**Charge**	**Spin**
A_1T_5	0.01	0.03	0.00	0.01	0.00	0.02	0.00
**Iz_2C_4**	0.01	0.04	0.00	0.03	0.00	0.03	0.00
A_3T_3	0.00	0.12	0.11	0.11	0.08	0.07	0.05
**$^OG_2C_4$**	-0.02	0.75	0.87	0.79	0.90	0.83	0.93
A_5T_1	0.00	0.06	0.02	0.06	0.02	0.05	0.02
T_1		-0.01	0.00	-0.02	0.00	-0.02	0.00
C_2		0.19	0.00	0.19	0.00	0.24	0.00
T_3		0.02	0.00	0.01	0.00	0.01	0.00
C_4		0.07	0.00	0.07	0.00	0.07	0.00
T_5		-0.02	0.00	-0.03	0.00	-0.03	0.00
A_1		0.05	0.00	0.04	0.00	0.05	0.00
Iz_2		-0.04	0.00	-0.04	0.00	-0.04	0.00
A_3		0.10	0.05	0.10	0.11	0.06	0.05
$^OG_2$		0.55	0.93	0.61	0.87	0.59	0.93
A_5		0.07	0.02	0.08	0.02	0.08	0.02
		**VA^{NE}**		**VA^{EQ}**		**AA**	
		Charge	Spin	Charge	Spin	Charge	Spin
A_1T_5		-0.10	0.05	-0.08	0.04	-0.05	0.02
**Iz_2C_4**		-0.78	0.92	-0.81	0.93	-0.84	0.97
A_3T_3		-0.06	0.03	-0.06	0.03	-0.10	0.01
**$^OG_2C_4$**		-0.04	0.00	-0.03	0.00	-0.01	0.00
A_5T_1		-0.02	0.00	-0.01	0.00	0.00	0.00
T_1		-0.03	0.00	-0.03	0.00	-0.02	0.00
C_2		0.15	0.00	0.15	0.00	0.15	0.00
T_3		-0.02	0.00	-0.01	0.00	-0.03	0.00
C_4		0.02	0.00	0.03	0.00	-0.05	0.00
T_5		-0.06	0.01	-0.05	0.00	-0.04	0.00
A_1		-0.04	0.04	-0.03	0.04	-0.01	0.02
Iz_2		-0.80	0.92	-0.84	0.93	-0.79	0.97
A_3		-0.04	0.03	-0.05	0.03	-0.08	0.01
$^OG_2$		-0.19	0.00	-0.18	0.00	-0.16	0.00

A_5		0.01	0.00	0.02	0.00	0.03	0.00
oligo-Oz							
	**Neutral**	**VC^{NE}**		**VC^{EQ}**		**AC**	
	**Charge**	**Charge**	**Spin**	**Charge**	**Spin**	**Charge**	**Spin**
A_1T_5	-0.02	-0.01	0.00	-0.02	0.00	0.01	0.00
**Oz_2C_4**	-0.01	0.02	0.00	0.01	0.00	0.00	0.00
A_3T_3	-0.01	0.10	0.08	0.08	0.06	0.10	0.06
**$^OG_2C_4$**	0.02	0.81	0.89	0.86	0.92	0.83	0.92
A_5T_1	0.01	0.08	0.02	0.07	0.02	0.06	0.02
T_1	-0.02	0.00	0.00	-0.02	0.00	-0.02	0.00
C_2	0.16	0.20	0.00	0.20	0.00	0.24	0.00
T_3	-0.03	0.00	0.00	-0.01	0.00	0.03	0.00
C_4	0.00	0.01	0.00	0.01	0.00	-0.02	0.00
T_5	-0.03	-0.02	0.00	-0.03	0.00	-0.02	0.00
A_1	0.01	0.02	0.00	0.01	0.00	0.03	0.00
Oz_2	-0.01	0.01	0.00	0.00	0.00	0.01	0.00
A_3	0.02	0.10	0.08	0.09	0.06	0.07	0.06
$^oG_2$	-0.14	0.61	0.89	0.66	0.92	0.58	0.92
A_5	0.04	0.08	0.02	0.09	0.02	0.08	0.02
		**VA^{NE}**		**VA^{EQ}**		**AA**	
		Charge	Spin	Charge	Spin	Charge	Spin
A_1T_5		-0,13	0,06	-0,10	0,03	-0,19	0,10
**Oz_2C_4**		-0,79	0,89	-0,85	0,93	-0,75	0,86
A_3T_3		-0,08	0,05	-0,08	0,04	-0,04	0,03
**$^OG_2C_4$**		0.00	0.00	0.01	0.00	-0.02	0.00
A_5T_1		0.00	0.00	0.01	0.00	0.00	0.00
T_1		-0.03	0.00	-0.02	0.00	-0.03	0.00
C_2		0.16	0.00	0.16	0.00	0.15	0.00
T_3		-0.05	0.01	-0.03	0.00	-0.03	0.03
C_4		-0.06	0.05	-0.04	0.02	-0.67	0.85
T_5		-0.07	0.02	-0.05	0.01	-0.15	0.10
A_1		-0.06	0.04	-0.05	0.03	-0.04	0.01
Oz_2		-0.73	0.84	-0.82	0.91	-0.07	0.01
A_3		-0.04	0.04	-0.04	0.04	-0.01	0.00
$^oG_2$		-0.15	0.00	-0.15	0.00	-0.17	0.00
A_5		0.03	0.00	0.04	0.00	0.02	0.00

Table S6a. The Energies: Ground (E^{GR}) and Excitation (E^{EX}) state energies and Excitation and HOMO Energies as well as corresponding Dipole Moments Ground, Excitation, and Transition (DM^{G}, DM^{EX}, D_{12}) in Debays of neighbor base pair extracted from selected dimmers of *ds*-oligonucleotides, calculated at the M06-2x/6-31++G** level of theory in the aqueous phase using the DFT or TD-DFT methodology.

**SYSTEM**	**B.P. Dimer**	E^{GR}	DM^{GR}	E^{EX}	DM^{EX}	D_{12}	E^{HOMO}	E^{HOMO-1}	E^{LUMO}	E^{LUMO+1}
oligo-Iz	$A_1 \Vert Iz_2$	-1726.906887	6.61	-1726.788562	7.23	0.73	-0.2793	-0.2965	-0.0462	-0.0169
	$Iz_2 \Vert A_3$	-1726.907658	7.56	-1726.789043	8.45	1.03	-0.2781	-0.2964	-0.0470	-0.0141
	$A_3 \Vert {}^{oxo}G_4$	-1933.69573	16.86	-1933.570352	15.20	3.50	-0.2515	-0.2837	-0.0157	-0.0152
	${}^{oxo}G_4 \Vert A_5$	-1933.693622	16.38	-1933.566204	14.77	4.35	-0.2546	-0.2840	-0.0176	-0.0138
oligo-Oz	$A_1 \Vert Oz_2$	-1895.555617	13.84	-1895.442378	15.37	0.37	-0.2842	-0.2961	-0.0735	-0.0175
	$Oz_2 \Vert A_3$	-1895.557571	14.43	-1895.444388	16.73	1.23	-0.2814	-0.2971	-0.0728	-0.0147
	$A_3 \Vert {}^{oxo}G_4$	-1933.696352	16.90	-1933.571578	15.24	3.29	-0.2512	-0.2833	-0.0163	-0.0151
	${}^{oxo}G_4 \Vert A_5$	-1933.693705	16.39	-1933.566309	14.79	1.74	-0.2545	-0.2838	-0.0178	-0.0139

Table S6b. The Energies: Ground (E^{GR}) and Excitation (E^{EX}) state energies and Excitation and HOMO Energies as well as corresponding Dipole Moments Ground, Excitation, and Transition (DM^{G}, DM^{EX}, D_{12}) in Debays of distal base pair extracted from selected trimmers of *ds*-oligonucleotides, calculated at the M06-2x/6-31++G** level of theory in the aqueous phase using the DFT or TD-DFT methodology

**SYSTEM**	**Base Pair Dimer**	E^{GR}	DM^{GR}	E^{EX}	DM^{EX}	D_{12}	E^{HOMO}	E^{HOMO-1}	E^{LUMO}	E^{LUMO+1}
oligo-Iz	$A_1 \Vert A_3$	-1726.906887	6.606798	-1726.788562	7.23	0.72	-0.279303	-0.296481	-0.046234	-0.016926
	$Iz_2 \Vert G_4$	-1895.557571	14.42834	-1895.444388	16.74	1.23	-0.281432	-0.29705	-0.072813	-0.014694
	$A_3 \Vert A_5$	-1933.696352	16.90233	-1933.571578	15.24	3.67	-0.251187	-0.283261	-0.016303	-0.015124
oligo-Oz	$A_1 \Vert A_3$	-1803.299495	7.075408	-1803.166029	7.39	**7.43**	-0.278048	-0.293616	-0.023881	-0.016382
	$Oz_2 \Vert G_4$	-1803.299996	8.358647	-1803.167826	8.69	**2.82**	-0.276475	-0.299718	-0.02411	-0.018991
	$A_3 \Vert A_5$	-1933.69568	16.21205	-1933.569391	14.48	**4.72**	-0.252935	-0.281799	-0.017415	-0.011527

Figure S1. The graphical representation of the ONIM layers distribution was used in these studies. Stick represented the sugar-phosphate backbone (the skeleton of a double helix) which has been described as a low-level layer and optimized on the M06-2x/sto-3G level of theory in the aqueous phase. Sticks and balls represented the High-level layer (base pairs, the ladder of double helix) which has been described as a low-level layer and optimized on the M06-2x/D95** level of theory in the aqueous phase.

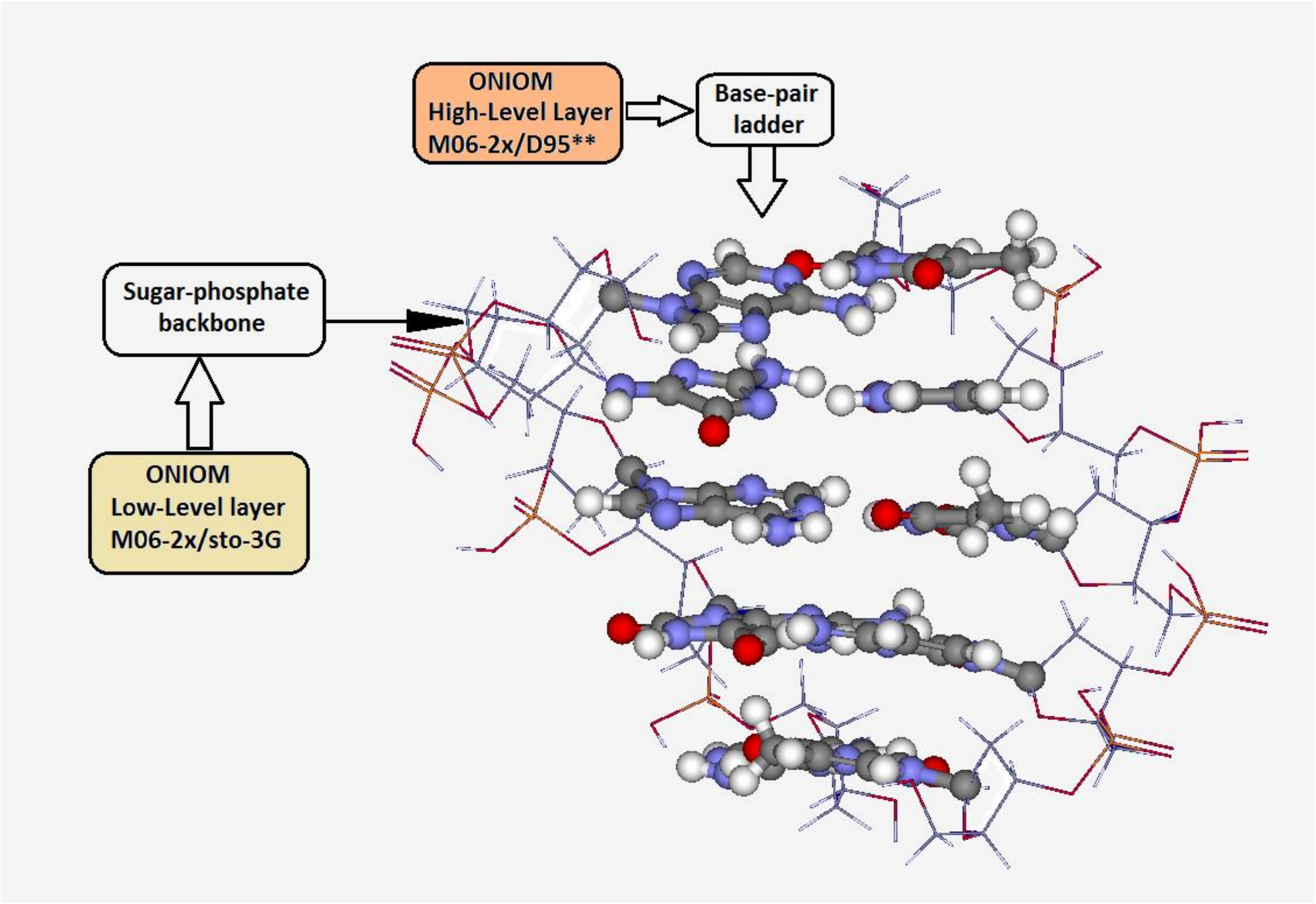

Supplement: Supplementary file 1 [file molecules-29-02754-s001.zip › molecules-3021757-supplementary.pdf]
